# GENETIC RISK FOR ADHD ON LATER LIFE EMOTIONAL AND COGNITIVE HEALTH: TESTING MECHANISMS OF EFFECT

**DOI:** 10.1093/geroni/igac059.1691

**Published:** 2022-12-20

**Authors:** Thalida Arpawong, Jimi Huh

**Affiliations:** University of Southern California, Los Angeles, California, United States; University of Southern California, Los Angeles, California, United States

## Abstract

Attention Deficit Hyperactivity Disorder (ADHD) is associated with emotional regulation and cognitive processing challenges throughout life. Not well-understood is how ADHD genetic risk (ADHD-GR) affects depressive symptoms (DepSx) and cognitive functioning in older age. Furthermore, less is known about the mechanisms through which some individuals show better outcomes despite higher ADHD-GR. We evaluated potential mechanisms using the Health and Retirement Study with data from 7,871 European Americans (EAs) and 1,226 African Americans (AAs), ages 50-93, and ADHD-GR calculated from a mixed ancestry genomewide scan. Mediators included closeness of parental relationships during childhood, sense of purpose in life in adulthood, and educational attainment. Outcomes included validated scales for DepSx and cognitive functioning. Structural equation models were stratified by race/ethnicity due to potential differences in genetic effects, adjusted for age, gender, ancestry, and health conditions with DepSx and genetic risk for dementia with cognitive functioning. Among EAs, ADHD-GR significantly predicted lower sense of purpose (p<.05) and worse parental relationships (p<.001), with higher levels of both, in turn, predicting less DepSx (p's< 0.01). ADHD-GR also showed significant direct effects on DepSx (p<.01), controlling for mediators. Additionally, ADHD-GR predicted less education (p<.001), but more education predicted better cognitive functioning (p< 0.001). No relationships with ADHD-GR were significant among AAs. Findings imply that among EAs, mediators could be targets to mitigate negative effects on psychological and cognitive health, two hallmark challenges for individuals with ADHD. More work is needed to characterize ADHD-GR on outcomes among older AAs.

